# Planting Material of Enset (*Ensete ventricosum*), a Key Food Security Crop in Southwest Ethiopia, Is a Key Element in the Dissemination of Plant-Parasitic Nematode Infection

**DOI:** 10.3389/fpls.2021.664155

**Published:** 2021-07-09

**Authors:** Selamawit A. Kidane, Solveig Haukeland, Beira H. Meressa, Anne Kathrine Hvoslef-Eide, Danny L. Coyne

**Affiliations:** ^1^Norwegian University of Life Sciences, Ås, Norway; ^2^International Institute of Tropical Agriculture, PMB, Ibadan, Nigeria; ^3^The Norwegian Institute of Bioeconomy Research, Ås, Norway; ^4^International Centre of Insect Physiology and Ecology, Nairobi, Kenya; ^5^Jimma University College of Agriculture and Veterinary Medicine, Jimma, Ethiopia; ^6^Nematology Research Unit, Department of Biology, Ghent University, Campus Ledeganck, Ledeganckstraat, Ghent, Belgium

**Keywords:** lesion nematode, *Pratylenchus goodeyi*, orphan crop, planting material quality, enset crop

## Abstract

Enset (*Ensete ventricosum*), is a perennial herbaceous plant belonging to the family Musaceae, along with banana and plantain. Despite wild populations occurring in eastern, central and southern Africa, it is only in Ethiopia that the crop has been domesticated, where it is culturally and agriculturally symbolic as a food security crop. Although an under-researched orphan crop, enset serves as a staple food for about 20% of the Ethiopian population, comprising more than 20 million people, demonstrating its value in the country. Similar to banana and plantain, enset is heavily affected by plant-parasitic nematodes, with recent studies indicating record levels of infection by the root lesion nematode *Pratylenchus goodeyi*. Enset is propagated vegetatively using suckers that are purposely initiated from the mother corm. However, while banana and plantain suckers have proven to be a key source of nematode infection and spread, knowledge on the infection levels and role of enset suckers in nematode dissemination is lacking. Given the high levels of plant-parasitic nematodes reported in previous surveys, it is therefore speculated that planting material may act as a key source of nematode dissemination. To address this lack of information, we assessed enset planting material in four key enset growing zones in Ethiopia. A total of 340 enset sucker samples were collected from farmers and markets and analyzed for the presence of nematodes. Nematodes were extracted using a modified Baermann method over a period of 48 h. The root lesion nematode *P. goodeyi* was present in 100% of the samples, at various levels of infection. These conclusive results show that planting material is indeed a key source of nematode infection in enset, hence measures taken to ensure clean suckers for planting will certainly mitigate nematode infection and spread. The effect of nematode infection on yield and quality on enset remains to be investigated and would be a way forward to complement the nematode/disease studies conducted so far and add valuable knowledge to the current poorly known impact of pests and diseases.

## Introduction

Described as the “tree against hunger” (Costa and Lockhart, 1984), enset (*Ensete ventricosum*) is a perennial monocarpic single-stemmed herbaceous plant belonging to the family Musaceae, along with banana and plantain. Although wild species occur in eastern, central and southern Africa (Baker and Simmonds, 1953) enset is cultivated in, and solely unique to, Ethiopia, where it is culturally and agriculturally symbolic; cropping systems in the south and southwest are based around this pivotal, yet under-researched orphan crop. Unlike bananas, enset does not produce edible fruits, instead, it is grown for its carbohydrate-rich food obtained from the pseudostem, leaf sheaths and underground corm, which are harvested and processed into food products. Harvest can be at any time during the year, at any growth stage and the fermented products can be stored for long periods, a combination of characters that make it an important food security crop, upon which millions depend. Its value was prominently highlighted during the harsh Ethiopian famine in the 1980's when enset growing communities were unaffected by the calamity (Dessalegn, 1995). However, on a regular basis, approximately 20% of the Ethiopian population depends on enset as a key staple food crop, primarily in the south and southwestern part of the country (Borrell et al., 2019, 2020). Furthermore, it is used for several other purposes, such as animal feed, fibre, construction material and in traditional medicine. The crop best grows at cooler, higher altitudes and is found mostly between 1200–3100 m above sea level (Brandt et al., 1997).

Harvest commonly occurs after 4 to 6 years after transplanting, but there is variability in when plants are harvested, with indications as early as three years and up to twelve years (Brandt et al., 1997; Borrell et al., 2020). Enset is vegetatively propagated using suckers that are produced through a succession of growth stages. Unlike banana, it does not produce suckers aside the mother plant, instead suckers are purposely initiated from a mother corm, obtained from harvested plants, after cutting off the pseudostem and roots and removing the apical dominance. Corms are then buried in the ground, just below the surface, and from which multiple suckers sprout and develop. Depending on the genotype and the size of the corm, between 20–100 suckers will arise (Brandt et al., 1997). After approximately one year, these suckers are transplanted into a well-manured nursery and repeatedly replanted, up to four times, into increasingly wider spaced nurseries until the suckers are removed for use as planting material. Suckers aged two to four years are used for planting into the field, many of which are sold at designated local seedling markets each year between December and February (Olango et al., 2014). Farmers also raise their own suckers or exchange planting materials between themselves.

Similar to banana and plantain, enset is heavily affected by plant-parasitic nematodes (Coyne and Kidane, 2018). Several plant-parasitic nematodes are associated with enset, with the lesion nematode, *P. goodeyi*, considered the most important threat to the crop (Peregrine and Bridge, 1992; Bogale et al., 2004; Addis et al., 2006; Kidane et al., 2020). For banana and plantain, the use of infected planting material (suckers) represents a key source of nematode dissemination and the perpetuation of the problem. Farmers exchange planting materials, and this practice is responsible for the continuous distribution of nematodes to new fields. The use of healthy planting materials, therefore, is essential to arrest the spread of nematodes and prevent losses due to the pests. A range of techniques is used in order to create healthy planting materials, such as through the use of *in vitro* tissue cultured material, macro propagation and sucker sanitation by paring and hot water treatment (Tenkouano et al., 2006; Coyne et al., 2010). The use of clean and healthy banana and plantain planting material plays a crucial role in averting the spread of nematodes and other root-borne pests and diseases and the damage they cause, especially in smallholder farming systems, where expensive management strategies are not feasible (Coyne et al., 2006).

Given the sparse knowledge by farmers of nematodes, as well as the current high incidence and levels of *P. goodeyi* infection on enset (Kidane et al., 2020), it is speculated that, similar to banana and plantain, nematodes are being disseminated to newly planted farms through the use of infected enset suckers. To date, there appears to be no information available or studies conducted to assess the level of nematode infection of enset suckers. The current study was undertaken to assess the infection status of enset planting materials as a basis for developing suitable nematode management options.

## Materials and Methods

**Figure 1:**
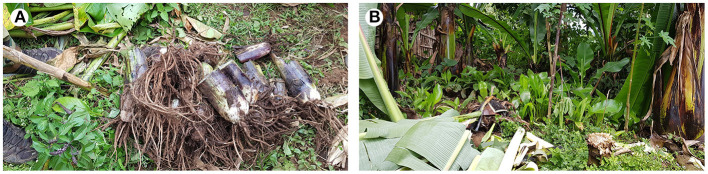
Enset suckers **(A) (B)** collected from farmers' nurseries in Ethiopia.

**Figure 2:**
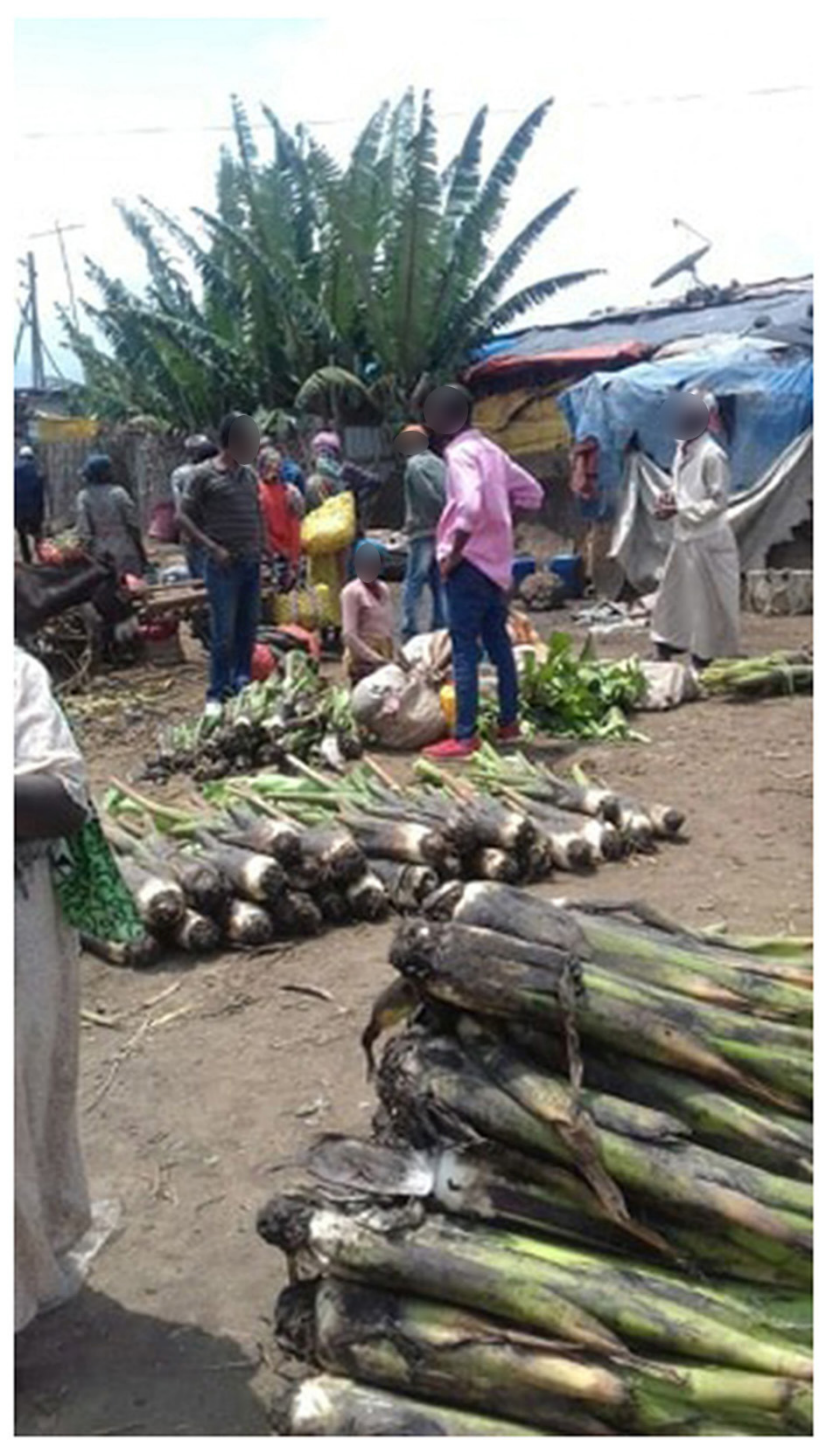
Enset suckers collected from markets in Ethiopia.

**Figure 3:**
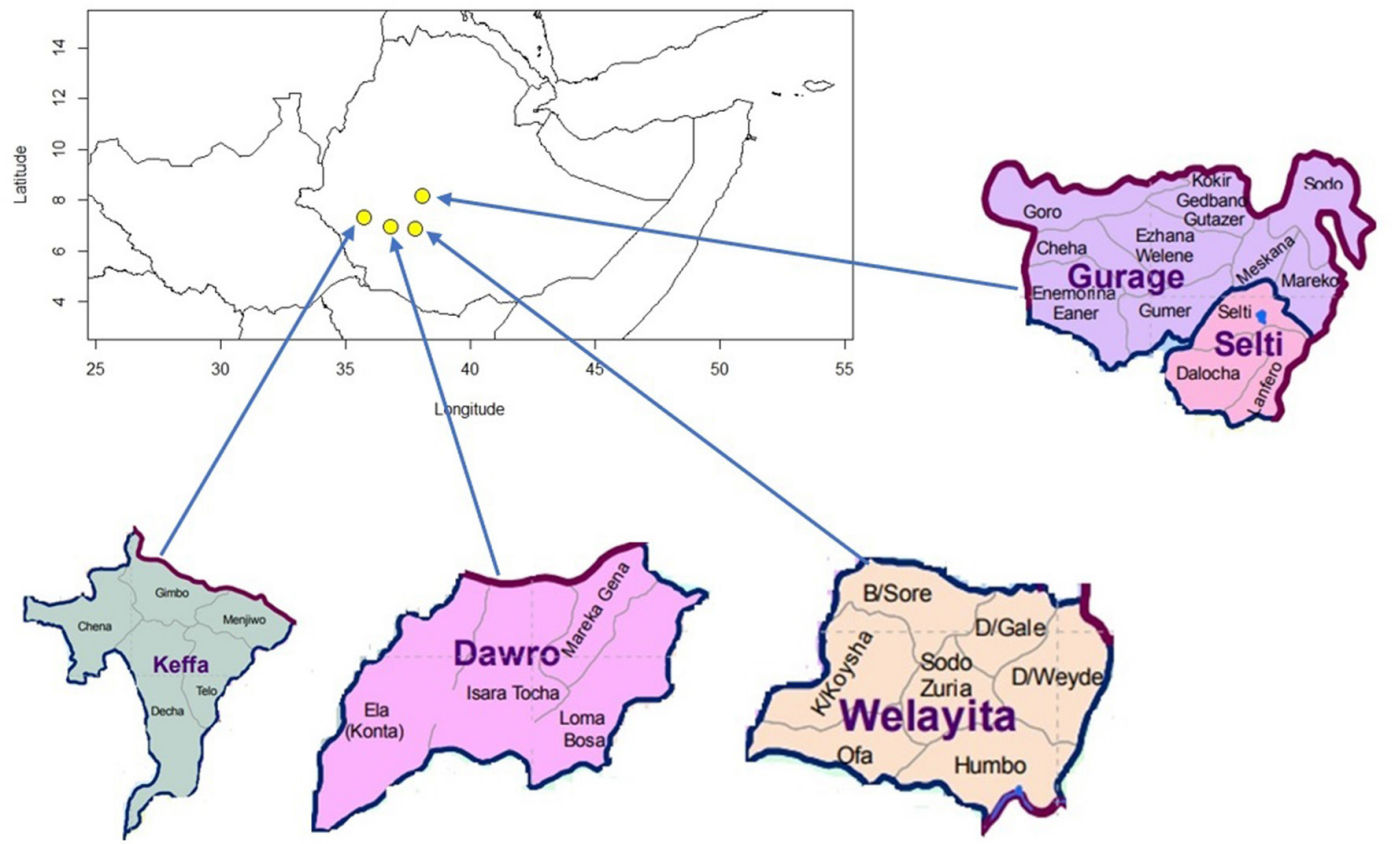
Enset growing zones in southern Ethiopia along with sites where enset suckers were collected.

Enset suckers aged between 1–2 years were collected from farmers (Figures 1A,B) and markets (Figure 2) in September–October 2019 in four key enset growing zones in Ethiopia (Dawro, Keffa, Guraghe and Wolayita) (Figure 3). In each of these growing zones, 13 locations were randomly selected and 16–40 enset suckers were collected at each site. The altitude was recorded for each site. The suckers were transported to the Plant Disease Diagnostics Lab of Jimma University, where roots were carefully washed, cut longitudinally, and chopped roughly into ~0.5 mm-size pieces and a 10 g sub-sample used for nematode extraction. Nematodes were extracted using a modified Baermann method over a period of 48 h (Coyne et al., 2018). Nematode suspensions were decanted, collected on a 38 μm sieve, rinsed into beakers, reduced to 10 ml and densities counted from 1 ml aliquots using a counting slide under a compound microscope. Nematode densities were calculated for each root sample and expressed as the number of nematodes per 10 g root. *Pratylenchus* specimens were identified to species level based on available keys (Sher and Allen, 1953; Castillo and Vovlas, 2007).

Nematode root density data were analyzed for any differences in infection levels between the regional zones. Each root sample was considered for analysis. All data were analyzed using R and RStudio® after log(n+1) transformation so that the data conformed to normal distribution (Zuur et al., 2010). The association between nematode density and altitude was analyzed using Pearson's correlation analysis.

## Results

**Figure 4:**
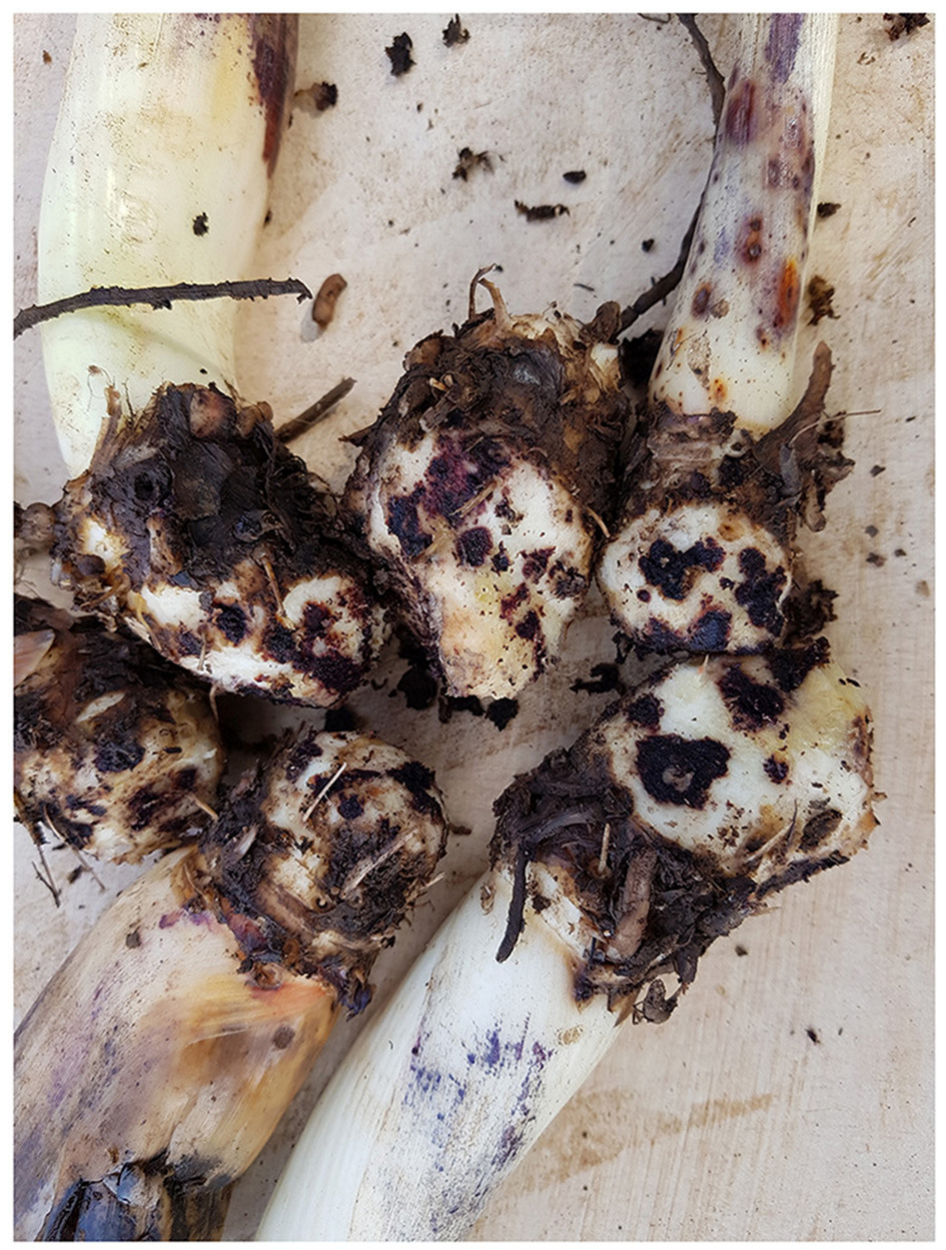
Extensive lesioning on enset suckers collected from markets and farms in Ethiopia.

A total of 340 enset samples each comprised of 2 to 3 suckers was assessed during the study. *P. goodeyi* was recovered from the roots of 100% of sucker samples which ranged in density from10 and 190 per 10 g roots (Table 1). Apart from a few non-parasitic nematodes in some samples, *P. goodeyi* was the only plant-parasitic nematode recovered from roots. Although the age of the suckers was not specifically recorded for each sample, in general younger suckers appeared less infected, than larger, older suckers (Kidane pers. obs.). On some suckers, especially the larger, older ones, lesions were clearly evident on their roots and corms (Figure 4).

**Table 1:** *Pratylenchus goodeyi* root density on enset sucker planting material collected from key enset production zones in Southern Ethiopia.

**Zone**	**Site/ elevation (m.a.s.l.)**	**Number of samples**	***Pratylenchus goodeyi* mean density per 10 g root**
Dawro	Tercha (1400)	24	141
	Maraka (2100–2200)	24	137
	Marimansa (1800–2000)	24	120
		Total = 72	
Keffa	Gimbo (1600–1900)	24	93
	Decha (1700–2100)	24	190
	Shishenda (1700–2200)	24	174
		Total = 72	
Guraghe	Ezha (> 2400)	25	140
	Meskan (2200)	22	70
	Abeshge (1600–1700)	16	69
	Silte (2000)	30	124
		Total = 93	
Wolayita	Boloso soro (1700–1800)	40	141
	Damot gale (2000)	40	10
	Sodo zuria (2000)	23	295
		Total = 103	

ANOVA revealed a significant difference (*P* < 0.05) in *P. goodeyi* root infection levels of sucker samples amongst sites. However, there was no correlation (*r* = 0.014; *P* = 0.85) in nematode infection with altitude, across all locations.

## Discussion

Infection of enset planting material with *P. goodeyi* is clearly widespread across the main enset growing zones in Ethiopia, and consequently acting as a key source of contamination of new fields. The nematode-infected suckers, often visibly affected with lesions on their roots and corms, are planted into new fields. Other than trimming the roots and parts of the corms, which is a common procedure performed during transplanting, there is no further treatment undertaken to reduce the nematode infection. With 100% infection incidence of planting material during the study, it is highly likely that this reflects the situation across all enset production systems in Ethiopia. Sucker infection levels were relatively high in some cases, and infection levels varied significantly amongst samples. This variability could be attributed to differences in susceptibility of the cultivars (Kidane et al., 2021), due to high genetic diversity among cultivated landraces (Kidane et al., 2020), each with varying levels of resistance against *P. goodeyi*. The current study aimed to assess the planting material most commonly available and used by farmers, which was suckers aged 1–2 years. However, when processing the suckers for nematode extraction, the older, larger suckers appeared to be relatively more infected, with more apparent lesions and damage observed in general (Figure 4). The variability in sucker age across samples may have additionally contributed to the high variability of nematode densities.

Interestingly, just one nematode pest species was recovered during the study. While several species of plant-parasitic nematodes are associated with enset in Ethiopia, *P. goodeyi* is the principal and most prevalent species (Bogale et al., 2004; Addis et al., 2006; Kidane et al., 2020). This is unlike other members of Musaceae, such as banana and plantain, for which several species often occur in combination (Coyne and Kidane, 2018; Sikora et al., 2018). As it appears that nematode pests are being constantly disseminated through contaminated planting material that is exchanged between farmers, the implementation of interventions that can avert this should be sought. Given the similarities with banana and plantain, experiences drawn from successful sucker sanitation practices in these crops, such as paring of corms and sucker immersion in boiling water for a brief 30 s prior to planting (Tenkouano et al., 2006), hold promise for enset.

In the current study, we observed that enset farmers had no perception of nematodes and the possible damage that they cause. This is despite a common practice of trimming necrotic sections from suckers before transplanting. Although the suckers are trimmed and cleaned to some degree, large amounts of necrotic tissue often remained on the transplanted suckers (Figure 3), indicating a lack of awareness of the importance of this damage by farmers. To date, there is no information available on the levels or extent of the damage being caused to enset production by *P. goodeyi*. It is effectively present in all plantations, to varying degrees of infection, but can be present at extremely high densities (Kidane et al., 2020). This blanket contamination of enset crops in Ethiopia has undoubted consequences to production and quality, which requires attention. Interventions to improve awareness of nematodes, the damage they cause, and suitable management strategies are required. However, the implementation of simple and effective options for the establishment of healthy seedling systems and sucker sanitation need to be prioritized. It is not surprising that a principal mode of nematode transmission on enset is through the dissemination of contaminated planting material. The current study confirms this and provides a basis for developing management options to amend this. Despite it being an important crop in various regards, the highly localized enset-based farming system has received only limited research attention, which needs to be rectified to ensure and improve the productivity of this neglected orphan crop (Brandt et al., 1997; Borrell et al., 2020).

## Data Availability Statement

The raw data supporting the conclusions of this article will be made available by the authors, without undue reservation.

## Author Contributions

SK planned, performed, analyzed and wrote the manuscript, SH helped planning the work and contributed to the writing, BM contributed to the planning, AH-E was the Project leader, contributed to the planning and contributed to the writing, DC planned, helped with the analysis and contributed to the writing of the manuscript. All authors contributed to the article and approved the submitted version.

## Conflict of Interest

The authors declare that the research was conducted in the absence of any commercial or financial relationships that could be construed as a potential conflict of interest.
